# Draft genome sequence of *Cronobacter dublinensis* S21, isolated from a child nutrition formula factory in Bangladesh

**DOI:** 10.1128/mra.00042-26

**Published:** 2026-05-05

**Authors:** Sutapa Bhowmik, Supantha Rivu, Md. Latiful Bari, Sangita Ahmed

**Affiliations:** 1 Department of Microbiology, Noakhali Science and Technology University 378872, Noakhali, Bangladesh; 2 Department of Microbiology, University of Dhaka 95324, Dhaka, Bangladesh; 3 Department of Microbiology, Notre Dame University Bangladesh 421903, Dhaka, Bangladesh; 4 Food, Nutrition and Agriculture Research Division, Centre for Advanced Research in Sciences, University of Dhaka 95324, Dhaka, Bangladesh; University of Manitoba, Winnipeg, Manitoba, Canada

**Keywords:** *Cronobacter dublinensis*, draft genome, food processing industry, Bangladesh

## Abstract

Here, we present the draft genome of *Cronobacter dublinensis* S21, isolated from a shoe rack at a child nutrition formula factory in Dhaka, Bangladesh. The genome is 4,564,625 bp with 4,085 predicted coding sequences and G+C content of 57.6%, and displays 40 virulence and 17 antibiotic resistance genes.

## ANNOUNCEMENT

*Cronobacter dublinensis* is a gram-negative bacterium belonging to the genus *Cronobacter* (1). Although no clinical cases of *C. dublinensis* have been reported to date, this bacterium has demonstrated a high capability to invade HEp-2 cells (2). *Cronobacter sakazakii*, another species within the genus, has been reported in several studies in Bangladesh (3–5); however, there is no information regarding *C. dublinensis* from this region. Here, we share the draft genome sequence of *C. dublinensis* S21, which was isolated in 2024 from a shoe rack at a child nutrition formula factory in Dhaka, Bangladesh.

A swab sample was collected from the shoe rack of a child nutrition formula factory in Dhaka (23°42′47.9808″ N, 90°34′36.1704″ E) and transferred immediately to the Department of Microbiology, University of Dhaka. The sample was enriched in Enterobacteria Enrichment Broth (Oxoid, England), followed by streaking on *Enterobacter sakazakii* agar (ESA) (Oxoid) and incubation at 37°C for 48 h (3). A blue colony from ESA was identified as a potential *Cronobacter* sp. microscopically. Later, *gluA* gene-specific polymerase chain reaction presumptively confirmed the isolate as *Cronobacter* sp. Using the Qiagen DNeasy Blood and Tissue Kit (250) (#69504; Qiagen, Hilden, Germany), genomic DNA was extracted from 1.5 mL of an overnight bacterial culture cultivated in Luria broth (Oxoid, Basingstoke, Hampshire, UK) at 37°C at 120 rpm. After ensuring the DNA quality using Nanodrop (ND-1000; Thermo Fisher Scientific, Waltham, MA, USA) and Qubit (Q33238, Thermo Fisher Scientific), it was sent to the International Center for Diarrheal Disease Research, Dhaka, Bangladesh, for whole-genome sequencing utilizing the Illumina NextSeq 2000 platform (Illumina, San Diego, CA, USA). For library preparation, the Illumina DNA Prep Reagent Kit (20060059) (Illumina) was used alongside an automated liquid handler (epMotion 5075) (3).

Upon assessing the quality using FastQC v0.12.1 (6), sequenced fastq files were trimmed using fastp v1.0.1 followed by assembly using SPAdes v4.2.0 (7, 8). Upon discarding contigs less than 500 bp, Prokka v1.14.6 was utilized for annotation (9). Virulence genes were detected using the Virulence Factor Database in ABRicate v1.0.1 on Galaxy with 80% coverage and 70% identity, respectively. Resistance Gene Identifier v6.0.5 in Comprehensive Antibiotic Resistance Database v4.0.1 was applied to detect antibiotic resistance genes (ARGs) (10–13). Later, PlasmidFinder v2.1.6, MobileElementFinder v1.0.3, and CRISPRCasFinder web server were used to detect plasmids, insertion sequences, and CRISPR arrays, respectively (14–16). All these tools were applied using default parameters unless otherwise specified.

**Fig 1:**
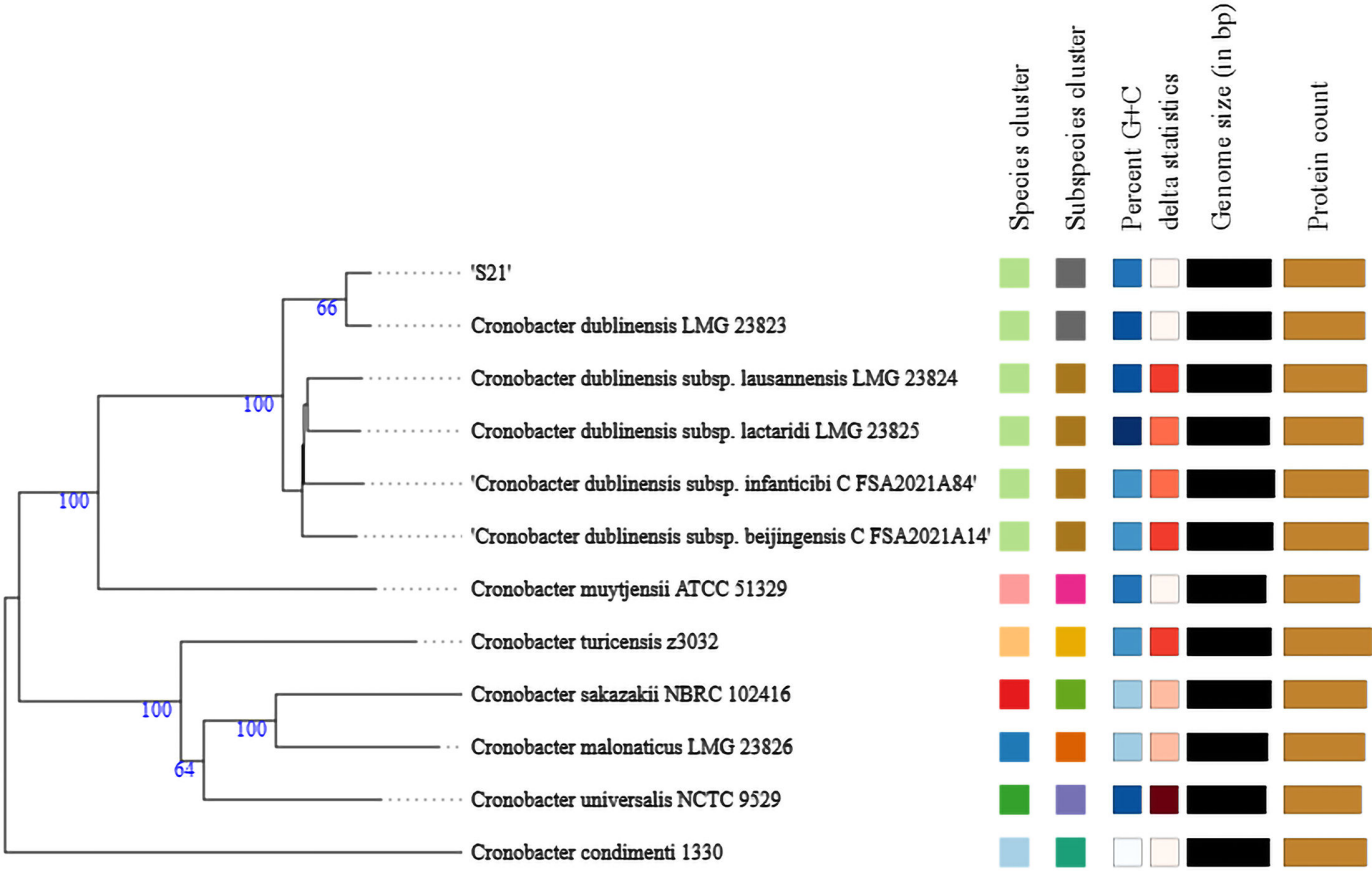
Phylogenetic analysis by Type Strain Genome Server shows that the study genome had the highest similarity with *C. dublinensis*. The whole-genome phylogenetic tree was constructed based on the Genome Blast Distance Phylogeny (GBDP) approach. It inferred with FastME version 2.1.6.1 (17) from GBDP distances calculated from genome sequences. The branch lengths are scaled in terms of GBDP distance formula d5. The numbers above branches are GBDP pseudo-bootstrap support values >60% from 100 replications, with an average branch support of 73.4%. The tree was rooted at the midpoint (18). Here, leaf labels are annotated by affiliation to species clusters, subspecies clusters, percent G+C, delta statistics, genome size (in bp), and protein count (from left to right).

The assembly consisted of 57 contigs with a total length of 4,564,625 bp and a G+C content of 57.6% (Table 1). The sequencing depth of the assembled genome was 51.23×. Whole-genome phylogenetic analysis using the Type Strain Genome Server grouped this genome with the *C. dublinensis* LMG 23823 (Fig. 1). Prokka revealed the presence of 4,085 coding DNA sequences, 67 tRNAs, and 3 rRNAs (Table 1). A total of 40 virulence genes, 17 ARGs, 1 plasmid, 2 insertion sequences, and 6 CRISPRs were detected in the genome (Table 1).

**TABLE 1:** Assembly statistics and important genomic features of *C. dublinensis* S21 from a shoe rack sample in Bangladesh

Parameter	Result for *C. dublinensis* S21
Sample source	Shoe rack
Sampling location	Child nutrition formula factory, Dhaka, Bangladesh
Sample type	Cotton swab
Year of isolation	2024
No. of reads	855,514
Total output (Mbp)	126.5
Type of reads	Paired end
Read length (bp)	35–151
Genome length (bp)	4,564,625
No. of contigs	57
GC content (%)	57.6
N50 (bp)	285,910
L50	7
Sequencing depth (×)	51.23
Coding DNA sequences	4,085
tRNAs	67
tmRNA	1
rRNAs	3
Antibiotic resistance genes	17 (*acrA*, *adeF*, *CRP*, *emrR*, *FosA8*, *H-NS*, *KpnE*, *KpnF*, *KpnH, marA*, *msbA*, *PBP3*, *qacG*, *rsmA, vanG*, *EF-Tu* mutants, *AcrAB-TolC* with *MarR* mutations)
Virulence genes	40 (*cheB*, *cheD*, *cheW*, *cheY*, *cheZ*, *csgD*, *csgF*, *csgG*, *entA*, *entB*, *entE*, *entS*, *fepA*, *fepB*, *fepC*, *fepD*, *fepG*, *flgB*, *flgC*, *flgG*, *flgH*, *flgI*, *flhA*, *flhC*, *fliA*, *fliG*, *fliI*, *fliM*, *fliP*, *fliS*, *hsiB1/vipA*, *hsiC1/vipB*, *htpB*, *IlpA*, *kdsA*, *kpsD*, *luxS*, *mgtB*, *motA*, *ompA*)
Plasmid	1 [Col(pHAD28) (KU674895)]
Insertion sequences	2 (ISEhe3, ISRaq1)
CRISPR array	6
Accession no.	NZ_JBSXVU000000000

## Data Availability

The whole-genome sequencing data for *C. dublinensis* S21 have been submitted to GenBank with accession no. NZ_JBSXVU000000000 under BioProject no. PRJNA1380941 (BioSample accession no. SAMN53932570 and SRA accession no. SRR37066346).
